# Effects of Different Dietary Starch Sources and Digestible Lysine Levels on Carcass Traits, Serum Metabolites, Liver Lipid and Breast Muscle Protein Metabolism in Broiler Chickens

**DOI:** 10.3390/ani13132104

**Published:** 2023-06-25

**Authors:** Caiwei Luo, Yanhong Chen, Dafei Yin, Jianmin Yuan

**Affiliations:** 1 State Key Laboratory of Animal Nutrition, College of Animal Science and Technology, China Agricultural University, Beijing 100193, China; 17330939818@163.com (C.L.); chenyanhong@cau.edu.cn (Y.C.); 2 College of Animal Husbandry and Veterinary Medicine, Shenyang Agricultural University, Shenyang 110866, China

**Keywords:** amylose, amylopectin, cassava, waxy corn, digestible lysine, broiler

## Abstract

**Simple Summary:**

In recent years, affected by the international trade situation, China’s corn import volume and import price have risen sharply, which has increased the production cost of poultry farming. Poultry nutritionists have been encouraged to use different grains instead of corn. However, it is unclear whether feeding broiler chickens with different starch sources affects the utilization of digestible lysine (**dLys**) in their body due to differences in digestion rate, thereby affecting broiler breast muscle protein and liver lipid metabolism. Therefore, this study aims at the above-mentioned problems. Here, we found that the waxy corn starch diet resulted in significantly higher expression levels of fat-synthesis-related genes than lipolysis-related genes, leading to abdominal fat deposition in broilers. Increasing the level of dLys in the diet increased the protein content in muscle by promoting protein synthesis and inhibiting protein degradation and also promoted the expression of lipolysis-related genes, thereby degrading the generation of abdominal fat in broilers. In conclusion, our findings signify that increasing the dLys level to 1.32% when using the waxy corn starch diet could improve carcass traits.

**Abstract:**

This study investigated the effects of digestible lysine (**dLys**) in different dietary starch sources on liver lipid metabolism and breast muscle protein metabolism in broiler chickens. The experimental design was a 3 × 3 two-factor completely randomized design. A total of 702 one-day-old male *Arbor Acres Plus* broilers were randomly divided into nine treatments of six replicate cages with thirteen birds each. The treatments consisted of three different starch sources (corn, cassava and waxy corn) with three different dLys levels (1.08%, 1.20% and 1.32%). The trial lasted from 1 to 21 days. Carcass traits, serum metabolites, breast muscle protein and liver lipid metabolism were evaluated. A significant interaction effect (*p* < 0.05) for dietary starch sources and dLys levels was noted in the percentage of abdominal fat and gene expression related to breast muscle protein metabolism throughout the experimental period. The waxy corn starch diet and a 1.08% dLys level in the diet increased both the percentage of abdominal fat (*p* < 0.01) and blood total cholesterol (*p* < 0.05) in the broilers. The waxy corn starch diet significantly upregulated the mRNA expressions of *Eif4E*, *AMPK*, *FABP1*, *ACC* and *CPT1* (*p* < 0.05). The 1.32% dLys level significantly upregulated the mRNA expressions of *mTOR*, *S6K1*, *Eif4E*, *AMPK* and *PPARα* (*p* < 0.05) and significantly downregulated the mRNA expressions of *MuRF* and *Atrogin-1* (*p* < 0.05). In summary, the waxy corn starch diet resulted in significantly higher expression levels of fat-synthesis-related genes than lipolysis-related genes, leading to abdominal fat deposition in broilers. Increasing the level of dLys in the diet increased the protein content in muscle by promoting protein synthesis and inhibiting protein degradation and also promoted the expression of lipolysis-related genes, thereby degrading the generation of abdominal fat in broilers. Our findings signify that increasing the dLys level to 1.32% when using the waxy corn starch diet could improve carcass traits.

## 1. Introduction

In recent years, affected by the international trade situation, corn import volume and import price have risen sharply in China, which has increased the production cost of poultry farming [1]. Therefore, poultry nutritionists have been encouraged to use alternative grains instead of corn. Grains mainly supply starch and serve as the primary source of energy. Starch includes two main components: amylose (**AM**) and amylopectin (**AP**). Previous reports have demonstrated that AP is easier to digest than AM [2], and starches with a higher ratio of AP may lead to a sharp rise in blood glucose and insulin levels [3]. Additionally, researchers found that the digestion kinetics of starch and amino acids are interdependent, with the properties of starch digestion affecting the metabolic pathways of amino acids [4]. When rapidly digestible starch (**RDS**, a low AM/AP starch) is consumed, glucose is rapidly released in the small intestine, a process that may not be able to continuously meet the normal energy requirements of broilers. In such cases, more amino acids may be oxidized to provide the energy needed by the body, resulting in a decrease in amino acid utilization [5].

Lysine (**Lys**) is defined as a basic essential amino acid whose carbon skeleton cannot be synthesized by poultry and therefore must be met through the diet for the maintenance and growth of poultry [6]. Lys is the second-most-limiting amino acid after methionine in corn–soybean meal-based diets for broilers. It is considered to be one of the main essential amino acids for muscle growth, development and deposition in broilers [7], especially for the turnover of breast muscle protein to regulate protein biosynthesis and decomposition [8,9]. At present, researchers have reported inconsistent results regarding the effect of dietary Lys on abdominal fat deposition in broilers. Maqsood et al. [10] believed that increasing dietary Lys levels could reduce the generation of abdominal fat in broilers. Tian et al. [11] found that a lack of dietary Lys can lead to a reduction in abdominal fat in broilers. However, the underlying mechanism by which dietary Lys directly or indirectly regulates breast protein and liver lipid metabolism in broilers is still unclear.

The synthesis of new muscle proteins is highly regulated by multiple signals integrated by the mammalian target of rapamycin (**mTOR**) [12]. In fact, *mTOR* promotes the synthesis of new proteins by directly phosphorylating the expression of translation-related downstream proteins, such as the signaling molecules ribosomal protein S6 kinase 1 (**S6K1**) and eukaryotic initiation factor 4E binding protein-1 (**Eif4E**) [13,14]. In addition, muscle protein content is also controlled by protein degradation, and protein degradation pathways are mainly divided into the ubiquitin–proteasome pathway (**UPP**) and autophagy–lysosome pathway [15]. The UPP is a critical protein breakdown pathway in eukaryotes, and scientists have demonstrated its importance in the muscle degradation process [16].

The liver is the center of glycogen synthesis, gluconeogenesis and energy metabolism in poultry. It acts as a central regulator of lipid homeostasis and is responsible for coordinating the synthesis, export and utilization of fatty acids as energy substrates [17,18]. Lipid metabolism in the body is mainly regulated by the adenosine 5`-monophosphate-activated protein kinase (**AMPK**) signaling pathway. The activation of *AMPK* inhibits lipogenesis and deposition while increasing fatty acid oxidation, affecting cholesterol and triglyceride synthesis, thereby regulating cellular energy balance [19]. A prior study noted that some genes such as peroxisome-proliferator-activated receptor α (**PPARα**), carbohydrate-responsive element-binding protein (**ChREBP**), sterol regulatory element-binding protein-1c (**SREBP-1c**), malic enzyme (**ME**) and acetyl CoA carboxylase (**ACC**) participate in hepatic lipid metabolism [11,20,21].

It is unclear whether feeding broiler chickens with different dietary starch sources would affect the utilization of dLys in their bodies, thereby affecting broiler breast muscle protein and liver lipid metabolism, due to differences in the digestion rates of these different starch sources. Therefore, the purpose of this study was to investigate the effects of different dLys levels on broiler carcass traits, serum metabolites and postprandial glucose and insulin changes under different dietary starch sources and to explore the responses of genes related to breast muscle protein and liver lipid metabolism to different starch sources and different dLys levels.

## 2. Materials and Methods

### 2.1. Ethics Statement

All animal procedures were conducted in accordance with the Beijing Regulations of Laboratory Animals (Beijing, China) and were approved by the Laboratory Animal Ethical Committee of China Agricultural University (Protocol Number: AW03602202-1-3).

### 2.2. Experimental Diets and Treatments

A total of 702 one-day-old male Arbor Acres Plus broiler chickens (from Beijing Poultry Breeding Company, Beijing, China) were randomly divided into 9 treatment groups based on a 3 × 3 two-factor experimental design. The treatments consisted of 3 different starch sources (corn, cassava and waxy corn) with 3 different dLys levels (1.08%, 1.20% and 1.32%). Each group included 6 replicate cages with 13 birds each. The experimental period was 21 days. The formula and nutritional level of the experimental diets are shown in Table 1.

### 2.3. Bird Husbandry

Bird management was based on the guide for *Arbor Acres Plus* broilers. All birds had access to feed and water ad libitum in crumble-pellet form and via nipple drinkers, respectively.

### 2.4. Sampling Procedures

On day 19, the birds from the dietary 1.20% dLys groups of different starch sources and the cassava starch groups of different dLys levels were selected. After fasting for 4 h, feeding for 0.5 h (set as zero) and at 0.5, 1.0, 1.5 and 2.0 h after feeding, blood samples were collected from birds’ wing veins in each group and kept in sterile serum collection tubes. Blood was collected once from each bird to avoid the influence of stress on the experimental result.

On day 21, six birds close to the average weight (one bird/replicate) in each treatment were selected for sample collection. The blood samples were drawn from birds’ wing veins and kept in sterile serum collection tubes. The birds were euthanized after intravenous injection of sodium pentobarbital (30 mg/kg). Six small pieces of the pectoralis and liver samples were washed with saline solution and collected into 1.5mL sterile Eppendorf tubes, and then snap-frozen in liquid nitrogen immediately and stored at −80 ℃ for mRNA analysis.

### 2.5. Carcass Characteristics Determination

On day 21, six birds close to the average weight (one bird/replicate) in each treatment were selected for slaughter, after which the breast muscle (%) and abdominal fat (%) were determined.

Breast muscle rate (%) = breast muscle weight/body weight × 100;

Percentage of abdominal fat (%) = abdominal fat weight/body weight × 100.

### 2.6. Blood Metabolite Analysis

The collected blood samples were centrifuged at 5000× *g* at 4 °C for 10 min, and the obtained serum samples were stored at −80 °C for later testing of glucose, cholesterol, triglycerides, uric acid and blood urea nitrogen. The serum glucose, insulin, urea nitrogen, uric acid, total cholesterol and triglycerides were analyzed using an XH-6080 radioimmunoassay analyzer.

### 2.7. Quantitative Real-Time PCR Analysis

The total RNA was extracted from the pectoralis and liver tissues using an RNAiso plus (Takara, Kyoto, Japan) according to the manufacturer’s recommendations. The nucleic acid concentration was determined using a Nanodrop 2000 spectrophotometer (Thermo Fisher Scientific, Waltham, MA, USA), and the RNA purity was verified using 1.5% denaturing agarose gels. Next, cDNA was synthesized by using a high-capacity cDNA reverse transcription kit (Takara, Kyoto, Japan) and stored at −20 °C. All quantitative real-time PCR (qRT-PCR) assays were performed using an SYBR Premix ExTap kit (Takara, Kyoto, Japan) in a 7500-fluorescence detection system (Applied Biosystems, Carlsbad, CA, USA). The primer sequences for qRT-PCR are listed in Table 2. All gene values were normalized to the expression of the housekeeping gene β-actin.

### 2.8. Statistical Analysis

The data were first tested for homogeneity of variances, after which the general linear model (**GLM**) of SPSS 20.0 statistical software (version 20.0, SPSS Inc., Chicago, IL, USA) was used to conduct a two-factor analysis of variance. The statistical model for this study is shown below. Differences between the treatment groups were considered statistically different at *p* < 0.05. All experimental data (carcass traits, serum metabolites, breast muscle protein metabolism and liver lipid metabolism) were based on one bird in each cage as the experimental unit. The different starch sources and different levels of digestible lysine were used as independent variables. The carcass characteristics, blood biochemistry, breast muscle protein and liver metabolic genes were used as dependent variables.

$$Y_{ijk}=\mu +\alpha_{i}+\beta_{j}+{\left(\alpha \times \beta \right)}_{ij}+\varepsilon_{ijk}$$

Here, *Y_ijk_* is the *k*th observation of the dependent variable recorded on the *i*th and *j*th treatments, *μ* is the overall mean, *α_i_* is the effect of the *i*th treatment, *β_j_* is the effect of the *j*th treatment, *(α × β)_ij_* is the interaction effect of the *i*th and *j*th treatments and *ε_ijk_* is the error associated with *Y_ijk_.*

## 3. Results

### 3.1. Carcass Traits

As shown in Table 3, there was no significant interaction effect between dietary starch sources and dLys levels on broiler breast muscle rates (*p* > 0.05). However, there was a significant interaction effect between dietary starch sources and dLys levels on the percentage of abdominal fat in broilers (*p* < 0.01). At the 1.08% and 1.32% dLys levels, waxy corn starch significantly increased the percentage of abdominal fat in the broiler chickens compared with corn starch (*p* < 0.01). At the 1.20% dLys level, different dietary starch sources had no significant effect on the abdominal fat percentage of the broilers (*p* > 0.05). In addition, increasing the level of dLys in waxy corn starch could also significantly reduce the percentage of abdominal fat in the broilers (*p* < 0.01).

### 3.2. Postprandial Blood Glucose and Insulin Responses

**Figure 1:**
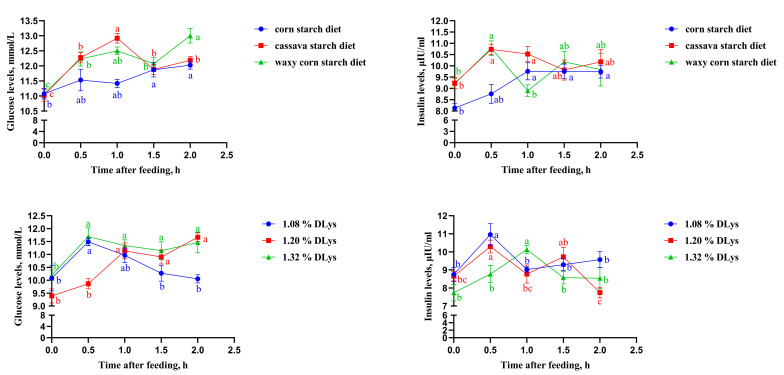
Effects of different starch sources and dLys levels on glucose and insulin responses in broiler chickens 2 h after feeding. ^a,b,c^ Means in the same color with different superscripts indicate differences or significant differences (*p* < 0.05).

The different starch sources and dLys levels affected the glucose and insulin responses in the broilers 2 h after feeding (Figure 1). At 0.5 h and 1.0 h after feeding, the cassava and waxy corn starches increased glucose levels more rapidly than the corn starch diet (*p* < 0.05). At 0.5 h after feeding, the insulin response level of the cassava and waxy corn starch diets reached their peaks, while the insulin response level of the corn starch diet increased steadily over time and finally remained at a stable level after 1.0 h.

The glucose levels in broilers subjected to 1.08% and 1.32% dLys peaked at 0.5 h after ingestion, but the glucose levels in broilers subjected to 1.32% dLys were relatively stable, and the glucose levels in broilers subjected to 1.08% dLys decreased rapidly, while the glucose levels in the broilers subjected to 1.20% dLys increased with time. In broilers subjected to 1.08% and 1.20% dLys, the insulin response levels reached a peak at 0.5 h after feeding, while the insulin response levels of broilers subjected to 1.32% dLys reached a peak at 1.0 h.

### 3.3. Serum Metabolites

The dietary starch sources and dLys levels had no significant interactions on serum urea nitrogen, uric acid, total cholesterol or triglyceride levels in the broilers (*p* > 0.05) (Table 4). The dietary starch sources and dLys levels had a significant effect on the serum total cholesterol in the broilers (*p* < 0.05). Compared with those consuming the corn starch diet, the serum total cholesterol levels of broilers consuming the cassava starch and waxy corn starch diets were significantly increased (*p* < 0.05). Compared with broilers subjected to 1.32% dLys, the serum total cholesterol levels in broilers in the 1.08% and 1.20% dLys groups were significantly increased (*p* < 0.01). However, the dietary starch sources and dLys levels had no significant effect on serum urea nitrogen, uric acid and triglyceride (*p* > 0.05).

### 3.4. Breast Muscle Protein Metabolism

There were significant interactions between dietary starch sources and dLys levels on the mRNA expressions of *S6K1*, *Eif4E*, *MuRF*, *CathepsinB*, *Atrogin-1* and *M-calpain* in broiler breast muscle (*p* < 0.05) (Table 5). At the 1.08% dLys level, cassava starch significantly increased the mRNA expression of *Eif4E* in breast muscle compared with corn starch and waxy corn starch (*p* < 0.05), and corn starch significantly increased the mRNA expressions of *MuRF*, *CathepsinB*, *Atrogin-1* and *M-calpain* in breast muscle compared with waxy corn starch (*p* < 0.05). At 1.20% dLys, cassava starch and waxy corn starch significantly increased the mRNA expression of *S6K1* and *Eif4E* in breast muscle compared with corn starch (*p* < 0.05), and waxy corn starch significantly increased the mRNA expression of *MuRF*, *CathepsinB*, *Atrogin-1* and *M-calpain* compared with cassava starch (*p* < 0.05). At the 1.32% dLys level, corn starch and waxy corn starch significantly increased the mRNA expression of *S6K1* and *Eif4E* in breast muscle compared with cassava starch (*p* < 0.05). DLys levels significantly affect the mRNA expression of *mTOR*: compared with 1.08% dLys, 1.32% dLys significantly increased the mRNA expression of *mTOR* (*p* < 0.05).

### 3.5. Liver Lipid Metabolism

Dietary starch sources and dLys levels showed no significant interactions with regard to the expression of genes related to lipid metabolism in the broiler liver (*p* > 0.05) (Table 6). Dietary starch sources had significant effects on the mRNA expressions of *AMPKα1*, *FABP1*, *ACC* and *CPT1* in broiler livers (*p* < 0.05): the waxy corn starch diet significantly increased the mRNA expressions of *AMPKα1*, *FABP1*, *ACC* and *CPT1* in broiler livers compared with the cassava and corn starch diets (*p* < 0.05). DLys levels significantly affect the mRNA expressions of *AMPKα1* and *PPARα* in the broiler liver (*p* < 0.01). Compared with 1.08% dLys, 1.20% and 1.32% dLys significantly increased the mRNA expressions of *AMPKα1* and *PPARα* (*p* < 0.01).

## 4. Discussion

Previous research found that Lys not only affected the process of body protein deposition and promoted the development of muscles (being especially important for the development of pectoral muscles) [22], but also regulated the expression of lipid synthesis genes and affected the deposition of fat in the body [11]. However, an unexpected result in this study was that dLys levels had no significant effect on broiler breast muscle growth. It is generally believed that broiler breast muscle growth increases with increasing Lys levels in the diet. Tesseraud et al. [9] proposed that Lys deficiency reduced muscle weights and protein contents by approximately −50% and −60% for the breast muscle and approximately −25% for the sartorius muscle. Tian et al. [11] found that broiler breast muscle growth with a Lys level of 0.60% in the diet was far lower than that of broilers with Lys levels of 1.00% and 1.40%. Based on these reports, we guessed that the insignificant results caused by adding different dLys levels in this study may be because the differences in dLys levels were too small and the broilers did not reach a state of deficiency or excess. Abdominal fat is an important indicator of fat deposition in broilers; excessive fat deposition is undesirable as it reduces feed efficiency and meat quality and increases production and health costs [23,24,25]. Previous entries in the literature have reported that higher Lys levels in the diet could help reduce the percentage of abdominal fat in broilers [10]. Similarly, this study also found that increasing dLys levels in the diet could reduce the percentage of abdominal fats of broilers fed with different dietary starch sources. To a certain extent, this shows that increasing dLys levels could reduce the waste of nutrients in the body, thereby promoting their distribution in the development of other tissues and organs, but the specific distribution method remains to be clarified by subsequent studies. In addition, this study also found that the percentage of abdominal fat in broilers in the waxy corn starch diet group was significantly higher than that of the other two groups. This is because a low AM/AP ratio diet induces faster and stronger blood glucose and insulin responses, resulting in the upregulation of genes involved in hepatic lipid synthesis and the increased activity of lipogenic enzymes [26,27], ultimately leading to abdominal fat deposition. In summary, we recommend that the dLys level in the diet should be appropriately increased when the waxy corn starch diet is used.

Uric acid is the end product of amino acid catabolism, and any amino acid not required as a building block during protein synthesis is broken down. During amino acid catabolism, the amino terminus of each amino acid must be removed. Because nitrogen compounds are not utilized in energy transduction pathways, the accumulation of amino groups may have adverse effects on the animal. Currently, researchers believe that serum uric acid levels can be used as a measure of amino acid availability [28] and, because uric acid levels in serum correlate with urinary and fecal nitrogen excretion rates, as a measure of nitrogen utilization. The formation of uric acid in the blood affects the efficiency of protein deposition in the muscles. In this study, it was found that, except for serum cholesterol, other serum metabolites were not affected by the dLys levels, which is consistent with the findings of Zarghi et al. [29]. At the same time, this is also consistent with the results of breast muscle growth in this study, because the breast muscle growth of each treatment group was not affected by the dLys levels. Cholesterol can be obtained from feed or synthesized in the body, and the synthesis rate of cholesterol is related to the lipid metabolism rate in the body. Studies by Emadi et al. [30] have shown that amino acid supplementation in diets could reduce blood cholesterol levels in broilers. This is consistent with the results of this study, where we observed that adding 1.32% dLys to broiler diets significantly reduced their serum cholesterol levels. This is also consistent with the results of abdominal fat production rates in this study, which demonstrate that the serum cholesterol level was positively correlated with the percentage of abdominal fat.

The amount of native starch hydrolyzed by amylase in the animal gut is negatively correlated with the dietary AM content [31]. Currently, high AM has been shown to differ from high AP in postprandial serum glucose and insulin responses and is associated with a lower rate of amylolysis [32,33]. The same phenomenon was also observed in the present study, where we determined the effects of corn, cassava and waxy corn starch diets on serum glucose and insulin changes in broilers within 2 h after feeding. As expected, the cassava and waxy corn starch diets contained higher levels of AP, which promoted faster and greater serum glucose and insulin responses in the body. This is in the same trend as the results of Ma et al. [34], and researchers have also observed similar phenomena in mammals [35,36]. In addition, we also observed that increasing dLys levels in the diet helped maintain the stability of glucose in the body. This may be because there is a certain interaction effect between higher dLys levels in the diet and glucose in the intestine, thereby promoting the continuous transfer of glucose in the intestine to the blood.

Amino acids play a key role in regulating protein synthesis or degradation in various tissues, including skeletal muscle. A study by Watanabe et al. [37] confirmed that feeding a low-Lys diet can significantly increase the mRNA expression of protein-degradation-related genes in broiler muscle. Li et al. [38] found that leucine stimulated *mTOR* signal passage and affected skeletal muscle protein synthesis. Similarly, Zheng et al. [39] also found that leucine could activate the *mTOR* signaling pathway, thereby enhancing the phosphorylation expression of its downstream signaling molecules *S6K1* and *Eif4E* and finally promoting protein synthesis in dairy cow skeletal muscle. *S6K1* mainly accelerates protein synthesis by enhancing translation initiation factors [40], while *Eif4E* promotes protein synthesis by stimulating the translation of a subset of mRNAs with 5`-terminal pyrimidine motifs [41]. Therefore, we speculated that the increase in muscle protein synthesis caused by Lys is also mediated by enhancing the translation initiation of its related mRNAs [42]. In the current study, we found that increasing the levels of dLys in the diet could significantly upregulate the expression of protein-synthesis-related genes such as *mTOR*, *S6K1* and *Eif4E*, which confirmed our speculation that increases in muscle protein synthesis caused by Lys are also mediated by enhancing the translation initiation of its related mRNAs. In addition, this study also found that a 1.32% dLys level could down-regulate the expression of protein-degradation-related genes such as muscle RING finger protein (**MuRF**) and muscle atrophy factor 1 (**Atrogin-1**). *Atrogin-1*, muscle atrophy F-box protein (**MAFbx**) and *MuRF* are the most representative E3 ubiquitin ligases in skeletal muscle, which mediate the polyubiquitination of proteins and target them to the 26S proteasome for degradation [43]. Currently, the expression of *MAFbx*, *MuRF* and *Atrogin-1* has been found to be significantly upregulated in various conditions that lead to muscle degradation [44]. Although there were no differences in breast muscle deposition in the current study, it increased numerically with increasing dLys levels. This further illustrated that Lys caused the enhancement of protein deposition in broiler muscle by promoting protein synthesis and inhibiting protein degradation.

As the main transcription factors in the *AMPK* signaling pathway, *PPARα* and *SREBP-1c* have been shown to play key roles in liver lipid metabolism [45]. In this study, we found that increasing dietary dLys levels significantly upregulated the mRNA expression of *AMPK* and *PPARα* in the liver tissue of broilers. *PPARα* activation induces the transcriptional upregulation of fatty acid transporters and downregulation of fatty acid synthesis [46]. Similarly, Sugden et al. [47] also showed that *PPARα* enhanced fatty acid β-oxidation by regulating the expression of *PPARα* target genes, thereby reducing the accumulation of lipids in vivo. In summary, increasing dietary dLys levels could activate the *AMPK* signaling pathway, thereby promoting the transcription level of *PPARα*, and finally inhibiting the accumulation of abdominal fat in broilers. Furthermore, the intake of a high AP diet can rapidly elevate blood glucose content in broilers, and blood glucose can change the expression of related transcription factors in a series of tissues through the lipid synthesis pathway, thus directly affecting the expression of lipogenic genes [27]. In the present study, the waxy corn starch diet significantly upregulated the expression of *AMPK*, *CPT1*, *FABP1* and *ACC* transcription factors. Although the genes related to lipid synthesis and decomposition were significantly upregulated in the broilers fed with the waxy corn starch diet, combined with the previous phenotypic results, we judge that the rate of lipid synthesis was significantly higher than the rate of lipid decomposition, which eventually led to abdominal fat deposition in the broilers. Our findings further support the results of Yang et al. [27] and Yin et al. [36].

## 5. Conclusions

In the current study, the waxy corn starch diet and a 1.08% dLys level in the diet increased both the percentage of abdominal fat and serum total cholesterol in broilers. The waxy corn starch diet resulted in significantly higher expression levels of fat-synthesis-related genes than lipolysis-related genes, leading to abdominal fat deposition in the broilers. Increasing dLys in the diet increased protein content in the muscles of the broilers by promoting protein synthesis and inhibiting protein degradation and also promoted the expression of lipolysis-related genes, thereby degrading the generation of abdominal fat in the broilers. In summary, our findings signify that increasing dLys levels to 1.32% when using the waxy corn starch diet could improve carcass traits.

## Figures and Tables

**Table 1:** Ingredient and nutrient composition of the experimental diet (%, as-fed basis).

Starch Sources	Corn			Cassava			Waxy Corn		
DLys Levels	1.08	1.20	1.32	1.08	1.20	1.32	1.08	1.20	1.32
Corn	40.70	41.60	42.60	40.70	41.60	42.60	40.70	41.60	42.60
Corn starch	15.00	15.00	15.00	-	-	-	-	-	-
Cassava starch	-	-	-	15.00	15.00	15.00	-	-	-
Waxy corn starch	-	-	-	-	-	-	15.00	15.00	15.00
Corn gluten meal	4.57	5.00	5.00	4.57	5.00	5.00	4.57	5.00	5.00
Soybean meal	33.23	31.30	29.80	33.23	31.30	29.80	33.23	31.30	29.80
Soybean oil	1.60	1.40	1.10	1.60	1.40	1.10	1.60	1.40	1.10
Calcium phosphate	1.67	1.70	1.70	1.67	1.70	1.70	1.67	1.70	1.70
Limestone	1.15	1.15	1.15	1.15	1.15	1.15	1.15	1.15	1.15
Sodium chloride	0.20	0.20	0.15	0.20	0.20	0.15	0.20	0.20	0.15
Sodium bicarbonate	0.33	0.33	0.38	0.33	0.33	0.38	0.33	0.33	0.38
Vitamins premix ^1^	0.03	0.03	0.03	0.03	0.03	0.03	0.03	0.03	0.03
Mineral premix ^2^	0.20	0.20	0.20	0.20	0.20	0.20	0.20	0.20	0.20
Choline chloride (50%)	0.16	0.16	0.16	0.16	0.16	0.16	0.16	0.16	0.16
Antioxidant	0.02	0.02	0.02	0.02	0.02	0.02	0.02	0.02	0.02
Phytase 10,000 U/g	0.02	0.02	0.02	0.02	0.02	0.02	0.02	0.02	0.02
L-Lysine hydrochloride (98%)	0.27	0.47	0.67	0.27	0.47	0.67	0.27	0.47	0.67
DL-Methionine (98%)	0.22	0.33	0.42	0.22	0.33	0.42	0.22	0.33	0.42
L-Threonine (98%)	0.06	0.16	0.26	0.06	0.16	0.26	0.06	0.16	0.26
L-Arginine hydrochloride (98%)	0.07	0.24	0.40	0.07	0.24	0.40	0.07	0.24	0.40
L-Tryptophan (98%)	-	-	0.03	-	-	0.03	-	-	0.03
L-Valine (98%)	-	0.08	0.20	-	0.08	0.20	-	0.08	0.20
Titanium dioxide	0.50	0.50	0.50	0.50	0.50	0.50	0.50	0.50	0.50
L-Isoleucine (98%)	-	0.11	0.21	-	0.11	0.21	-	0.11	0.21
Total	100.00	100.00	100.00	100.00	100.00	100.00	100.00	100.00	100.00
Nutritional Level									
Metabolizable energy (Mcal/kg) ^3^	3.02	2.99	2.97	3.00	2.91	2.94	2.95	3.00	2.94
CP, %	20.99	21.05	21.06	20.99	21.05	21.06	20.99	21.05	21.06
Calcium, %	0.95	0.95	0.94	0.95	0.95	0.94	0.95	0.95	0.94
NPP, %	0.37	0.37	0.37	0.37	0.37	0.37	0.37	0.37	0.37
Sodium, %	0.19	0.19	0.18	0.19	0.19	0.18	0.19	0.19	0.18
Chlorine, %	0.23	0.27	0.28	0.23	0.27	0.28	0.23	0.27	0.28
Digestible lysine, %	1.08	1.20	1.32	1.08	1.20	1.32	1.08	1.20	1.32
Digestible methionine, %	0.50	0.60	0.69	0.50	0.60	0.69	0.50	0.60	0.69
Digestible methionine + cystine, %	0.78	0.89	0.96	0.78	0.89	0.96	0.78	0.89	0.96
Digestible threonine, %	0.71	0.79	0.87	0.71	0.79	0.87	0.71	0.79	0.87
Digestible valine, %	0.84	0.90	0.99	0.84	0.90	0.99	0.84	0.90	0.99
Digestible arginine, %	1.16	1.28	1.4	1.16	1.28	1.4	1.16	1.28	1.4
Digestible isoleucine, %	0.74	0.83	0.90	0.74	0.83	0.90	0.74	0.83	0.90
Digestible leucine, %	1.72	1.71	1.68	1.72	1.71	1.68	1.72	1.71	1.68
Digestible tryptophan, %	0.21	0.20	0.22	0.21	0.20	0.22	0.21	0.20	0.22

Abbreviations: CP: crude protein; NPP: non-phytate phosphorus. ^1^ The vitamin premix provided (per kg of diet) the following: vitamin A, 15,000 IU; vitamin D3, 3600 IU; vitamin E, 30 IU; vitamin K3, 3.00 mg; vitaminB2, 9.60 mg; vitamin B12, 0.03 mg; biotin, 0.15 mg; folic acid, 1.50 mg; pantothenic acid, 13.80 mg; nicotinic acid, 45 mg. ^2^ The trace mineral premix provided (per kg of diet) the following: Cu, 16 mg; Zn, 110 mg; Fe, 80 mg; Mn, 120 mg; Se, 0.30 mg; I, 1.50 mg. ^3^ Analysis values of nutrient components.

**Table 2:** Primer sequences of RT-PCR.

Gene	Forward Sequences (5′–3′)	Reverse Sequences (5′–3′)
*β-actin*	GAGAAATTGTGCGTGACATCA	CCTGAACCTCTCATTGCCA
*mTOR*	AGTGAGAGTGATGCGGAGAG	GAAACCTTGGACAGCGGG
*S6K1*	GGTGGAGTTTGGGGGCATTA	GAAGAACGGGTGAGCCTAA
*MAFbx*	CCTTCACAGACCTGCCATTG	GCAGAGCTTCTTCCACAGCA
*MuRF*	GCTGGTGGAGAACATCATCG	GCTGGTGGAGAACATCATCG
*Eif4E*	TGGAACCGGAAACCACTCCC	GCGCCCATCTGTTTTGTAGTG
*CathepsinB*	TGTGGAAGCGATTTCGGACA	TAACCACCATTGCACCCCAT
*Atrogin-1*	CAGACAGATTCGCAAACGGC	CTCCTTCCGTGGGTAACACC
*m-calpain*	TGGAAGCTGCAGGGTTCAAG	GGTTTCCAGCCGAATCAAGC
*AMPK*	ATCTGTCTCGCCCTCATCCT	CCACTTCGCTCTTCTTACACCTT
*FABP1*	CAGGAGAGAAGGCCAAGTGTA	TGGTGTCTCCGTTGAGTTCG
*PPARα*	AGAGCCACTTGCTATCACCA	GTCATTTCACTTCACGCAGCA
*ACC*	TGTTGAAGGTGACCCGACAG	AAGATAGGAGCAGCCCTCCA
*ME*	AATACACAGAGGGACGTGGC	GCAACTCCAGGGAACACGTA
*SREBP-1*	GCCATCGAGTACATCCGCTT	GGTCCTTGAGGGACTTGCTC
*CPT1*	GTGAGTGATTGGTGGGAAGA	CCTGTATGGTTGTGGGAGATAAA

Abbreviations: *mTOR*: mammalian target of rapamycin; *S6K1*: ribosomal protein S6 kinase 1; *Eif4E*: eukaryotic initiation factor 4E binding protein-1; *MAFbx*: muscle atrophy F-box; Atrogin-1: muscle atrophy factor-1; *MuRF*: muscle RING finger protein-1; *AMPK*: adenosine 5′-monophosphate-activated protein kinase; *FABP1*: fatty acid binding protein 1; SREBP-1: sterol regulatory element binding proteins-1c; *ACC*: acetyl CoA carboxylase; *ME*: malic enzyme; *PPARα*: peroxisome proliferators-activated receptors α; *CPT1*: carnitine palmitoyl transferase 1.

**Table 3:** Effects of different starch sources and dLys levels on carcass traits of 21 d broilers.

Item		Breast Muscle, %	Abdominal Fat, %
Corn starch	1.08% dLys	16.52	0.92 ^bc^
	1.20% dLys	16.26	0.88 ^cd^
	1.32% dLys	17.22	0.76 ^e^
Cassava starch	1.08% dLys	16.48	1.00 ^a^
	1.20% dLys	16.81	0.86 ^d^
	1.32% dLys	17.09	0.67 ^f^
Waxy corn starch	1.08% dLys	16.49	1.01 ^a^
	1.20% dLys	16.83	0.90 ^bcd^
	1.32% dLys	16.71	0.93 ^b^
SEM		0.16	0.01
Main effect			
Starch source	Corn starch	16.66	0.85 ^b^
	Cassava starch	16.79	0.84 ^b^
	Waxy corn starch	16.68	0.95 ^a^
dLys level	1.08%	16.50	0.98 ^a^
	1.20%	16.63	0.88 ^b^
	1.32%	17.01	0.79 ^c^
*p* value			
Starch source		0.948	<0.001
dLys level		0.503	<0.001
Starch source × dLys level		0.898	<0.001

^a–f^ Means in the same column with different superscripts indicate differences or significant differences (*p* < 0.05).

**Table 4:** Effects of different starch sources and dLys levels on blood biochemistry of 21 d broilers.

Item		Urea Nitrogen, mmol/L	Uric Acid, μmol/L	Total Cholesterol, mmol/L	Triglyceride, mmol/L
Corn starch	1.08% dLys	0.617	251.33	3.14	0.363
	1.20% dLys	0.783	206.33	3.13	0.335
	1.32% dLys	0.917	196.00	2.63	0.350
Cassava starch	1.08% dLys	0.683	215.33	3.20	0.322
	1.20% dLys	0.817	224.50	3.50	0.332
	1.32% dLys	0.783	229.50	3.13	0.363
Waxy corn starch	1.08% dLys	0.850	246.33	3.27	0.393
	1.20% dLys	0.667	196.83	3.49	0.377
	1.32% dLys	0.833	188.67	3.02	0.342
SEM		0.029	8.24	0.05	0.012
Main effect					
Starch source	Corn starch	0.772	217.89	2.97 ^b^	0.349
	Cassava starch	0.761	223.11	3.28 ^a^	0.339
	Waxy corn starch	0.783	210.61	3.26 ^a^	0.371
dLys level	1.08%	0.717	204.72	3.20 ^a^	0.359
	1.20%	0.756	209.22	3.37 ^a^	0.348
	1.32%	0.844	237.67	2.92 ^b^	0.352
*p* value					
Starch source		0.950	0.830	0.012	0.596
dLys level		0.180	0.230	0.001	0.932
Starch source × dLys level		0.163	0.569	0.568	0.791

^a,b^ Means in the same column with different superscripts indicate differences or significant differences (*p* < 0.05).

**Table 5:** Effects of different starch sources and dLys levels on breast muscle protein metabolism of 21 d broilers.

Item		*mTOR*	*S6K1*	*Eif4E*	*MAFbx*	*MuRF*	*CathepsinB*	*Atrogin-1*	*M-Calpain*
Corn starch	1.08% dLys	0.95	0.85 ^d^	0.66 ^b^	1.37	1.62 ^a^	1.46 ^a^	1.23 ^a^	1.28 ^a^
	1.20% dLys	0.88	0.86 ^d^	0.74 ^b^	1.10	0.85 ^b^	1.13 ^abc^	1.11 ^ab^	0.68 ^c^
	1.32% dLys	1.01	1.41 ^ab^	1.22 ^a^	0.69	0.70 ^b^	1.20 ^abc^	0.44 ^c^	0.98 ^bc^
Cassava starch	1.08% dLys	0.74	1.10 ^abcd^	1.16 ^a^	1.04	1.20 ^ab^	1.12 ^abc^	0.85 ^abc^	0.95 ^bc^
	1.20% dLys	0.76	1.42 ^ab^	1.11 ^a^	0.93	0.93 ^b^	1.00 ^c^	0.63 ^c^	0.82 ^bc^
	1.32% dLys	1.14	0.90 ^cd^	0.72 ^b^	1.25	0.93 ^b^	1.14 ^abc^	0.77 ^abc^	0.96 ^bc^
Waxy corn starch	1.08% dLys	0.72	0.97 ^bcd^	0.73 ^b^	0.86	0.93 ^b^	0.90 ^c^	0.80 ^abc^	0.71 ^c^
	1.20% dLys	1.07	1.33 ^abc^	1.30 ^a^	0.86	1.57 ^a^	1.36 ^ab^	1.16 ^ab^	1.04 ^ab^
	1.32% dLys	1.22	1.48 ^a^	1.28 ^a^	0.80	0.95 ^b^	1.31 ^ab^	0.74 ^bc^	0.75 ^bc^
SEM		0.04	0.06	0.05	0.08	0.07	0.04	0.06	0.04
Main effect									
Starch source	Corn starch	0.95	1.04	0.88 ^b^	1.05	1.06	1.26	0.93	0.98
	Cassava starch	0.88	1.14	1.00 ^ab^	1.07	1.02	1.08	0.75	0.91
	Waxy corn starch	1.00	1.26	1.10 ^a^	0.84	1.15	1.19	0.90	0.83
dLys level	1.08%	0.80 ^b^	0.98 ^b^	0.85 ^b^	1.09	1.25 ^a^	1.16	0.96 ^a^	0.98
	1.20%	0.90 ^b^	1.20 ^ab^	1.05 ^a^	0.96	1.12 ^ab^	1.16	0.97 ^a^	0.85
	1.32%	1.12 ^a^	1.26 ^a^	1.07 ^a^	0.91	0.86 ^b^	1.21	0.65 ^b^	0.89
*p* value									
Starch source		0.370	0.180	0.037	0.422	0.635	0.173	0.289	0.215
dLys level		0.002	0.044	0.021	0.650	0.029	0.792	0.014	0.245
Starch source × dLys level		0.134	0.006	<0.001	0.382	0.003	0.010	0.014	0.001

^a,b,c,d^ Means in the same column with different superscripts indicate differences or significant differences (*p* < 0.05). *mTOR*: mammalian target of rapamycin; *S6K1*: ribosomal protein S6 kinase 1; *Eif4E*: eukaryotic initiation factor 4E binding protein-1; *MAFbx*: muscle atrophy F-box; *Atrogin-1*: muscle atrophy factor-1; *MuRF*: muscle RING finger protein-1.

**Table 6:** Effects of different starch sources and dLys levels on liver lipid metabolism of 21 d broilers.

Item		*AMPK*	*FABP1*	*SREBP-1*	*ACC*	*ME*	*PPARα*	*CPT1*
Corn starch	1.08% dLys	0.54	0.40	1.82	0.64	1.43	0.45	0.40
	1.20% dLys	0.74	0.79	1.02	0.77	1.53	0.66	0.55
	1.32% dLys	0.70	0.62	1.42	1.30	2.29	1.28	0.49
Cassava starch	1.08% dLys	0.61	0.52	1.53	0.85	1.84	0.55	0.49
	1.20% dLys	0.80	0.59	2.23	1.46	1.69	0.72	0.56
	1.32% dLys	0.74	0.56	1.23	0.91	1.10	1.12	0.61
Waxy corn starch	1.08% dLys	0.75	0.78	1.74	1.12	1.75	0.71	0.76
	1.20% dLys	1.13	0.77	1.82	1.59	1.29	1.36	1.03
	1.32% dLys	1.32	0.84	2.31	1.86	1.47	1.16	0.69
SEM		0.05	0.04	0.11	0.11	0.11	0.07	0.05
Main effect								
Starch source	Corn starch	0.66 ^b^	0.60 ^b^	1.42	0.90 ^b^	1.75	0.79	0.48 ^b^
	Cassava starch	0.72 ^b^	0.55 ^b^	1.66	1.07 ^ab^	1.54	0.80	0.55 ^b^
	Waxy corn starch	1.06 ^a^	0.80 ^a^	1.96	1.52 ^a^	1.50	1.08	0.83 ^a^
dLys level	1.08%	0.63 ^b^	0.57	1.70	0.87	1.67	0.57 ^b^	0.55
	1.20%	0.89 ^a^	0.72	1.69	1.27	1.51	0.91 ^a^	0.71
	1.32%	0.92 ^a^	0.67	1.65	1.36	1.62	1.18 ^a^	0.60
*p* value								
Starch source		<0.001	0.031	0.127	0.047	0.614	0.071	0.025
dLys level		0.006	0.260	0.984	0.123	0.815	<0.001	0.433
Starch source × dLys level		0.343	0.486	0.051	0.460	0.122	0.168	0.803

^a,b^ Means in the same column with different superscripts indicate differences or significant differences (*p* < 0.05). *AMPK*: adenosine 5′-monophosphate-activated protein kinase; *FABP1*: fatty acid binding protein 1; *SREBP-1*: sterol regulatory element binding proteins-1c; *ACC*: Acetyl CoA carboxylase; *ME*: malic enzyme; *PPARα*: peroxisome proliferators-activated receptors α; *CPT1*: carnitine palmitoyl transferase 1.

## Data Availability

The data presented in this study are available on request from the corresponding author.
